# Exploration of thiaheterocyclic *h*HDAC6 inhibitors as potential antiplasmodial agents

**DOI:** 10.4155/fmc-2016-0215

**Published:** 2017-03-06

**Authors:** Rob De Vreese, Carmen de Kock, Peter J Smith, Kelly Chibale, Matthias D'hooghe

**Affiliations:** 1 SynBioC Research Group, Department of Sustainable Organic Chemistry & Technology, Faculty of Bioscience Engineering, Ghent University, Coupure Links 653, B-9000, Ghent, Belgium; 2 Division of Pharmacology, Department of Medicine, University of Cape Town, K45, OMB Groote Schuur Hospital, Observatory 7925, South Africa; 3 South African Medical Research Council Drug Discovery & Development Research Unit, Department of Chemistry & Institute of Infectious Disease & Molecular Medicine, University of Cape Town, Rondebosch 7701, South Africa

**Keywords:** benzohydroxamic acids, HDAC6, malaria, *Plasmodium falciparum*, thiaheterocycles

## Abstract

**Aim:**

The recurring resistance of the malaria parasite to many drugs compels the design of innovative chemical entities in antimalarial research. Pan-histone deacetylase inhibitors (pan-HDACis) have recently been presented in the literature as powerful novel antimalarials, although their application is hampered due to toxic side effects. This drawback might be neutralized by the deployment of isoform-selective HDACis.

**Results:**

In this study, 42 thiaheterocyclic benzohydroxamic acids, 17 of them being potent and selective *h*HDAC6 inhibitors, were tested to investigate a possible correlation between *h*HDAC6 inhibition and antiplasmodial activity.

**Conclusion:**

Four *h*HDAC6 inhibitors showed submicromolar potency against both a chloroquine-sensitive and a chloroquine-resistant strain of *Plasmodium falciparum* with high selectivity indices, pointing to the relevance of exploring *h*HDAC6 inhibitors as potential new antiplasmodial agents.

## Graphical abstract

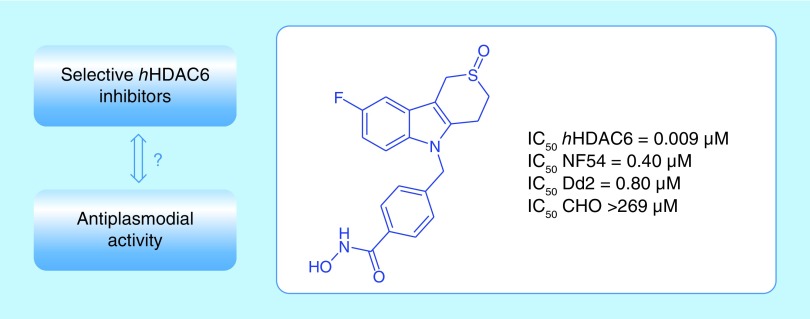

**Figure 1.**
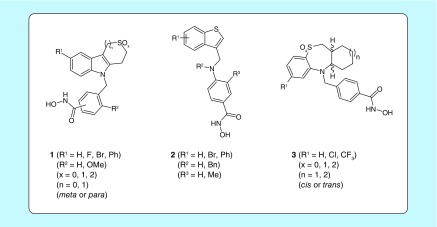
**Available thiaheterocyclic benzohydroxamic acids 1–3.**

First draft submitted: 16 November2016; Accepted for publication: 12 January 2017; Published online: 6 March 2017

Malaria is a devastating parasitic disease, exemplified by the fact that roughly 3.2 billion people are at risk of contracting malaria and that this disease caused roughly 438,000 deaths in 2015, with an estimated 306,000 casualties in the group of children under the age of five (WHO) [1]. The main culprit causing this infection is the protozoan species *Plasmodium falciparum*, transmitted by mosquitoes of the genus *Anopheles* [2]. In the past 15 years (2000–2015), considerable progress has been made toward revoking this infection, as illustrated by a declining number of malaria cases and deaths (18 and 48%, respectively) [1]. However, there still is a pressing need to reduce the number of victims even further and to find solutions to address all challenges associated with this disease. A pertinent challenge relates to the expanding resistance of the *Plasmodium* parasite toward several treatment regimes. Indeed, resistance has emerged with respect to the standard antimalarials chloroquine (CQ), sulfadoxine, pyrimethamine and, more recently, artemisinin [3]. The acquired artemisinin resistance is particularly alarming, since artemisinin combination therapies represent the first-in-line treatment option for malaria nowadays.

A consequence of this recurring resistance is the urgent need to develop new medicines with alternative mechanisms of action, in order to impede or deter the parasite from developing resistance by applying combination therapies. In that regard, histone deacetylase inhibitors (HDACis) might offer new treatment opportunities, as several known HDAC inhibitors have recently been shown to demonstrate a promising activity against *P. falciparum* and other malarial strains [4–7]. HDACs and HATs function as regulators of lysine acetylation, an important post-translational modification responsible for the neutralization of the positive charges on lysine residues, and as such adjusting the exact mode of action of the targeted protein [8]. HDACs were first been discovered as histone lysine modifying enzymes but are now generally accepted to be lysine deacetylases, also deacetylating several nonhistone proteins [9]. In humans, this group of enzymes comprises four classes, with class I (HDAC1, 2, 3 and 8), IIa (HDAC4, 5, 7 and 9), IIb (HDAC6 and 10) and IV (HDAC11) employing zinc as an essential cofactor, while class III (SIRT1–7) uses NAD^+^ for its deacetylase activity [10]. On the other hand, five HDAC isoforms are known for *P. falciparum*: P*f*HDAC1, with homology to human class I, P*f*HDAC2 and 3, with homology to human class II, and *Pf*Sir2A and *Pf*Sir2B, with homology to human class III [11]. So far, mainly pan-HDACi's have been tested for their activity against *P. falciparum*, revealing high toxicities in the (low) nanomolar range toward the malaria parasite [4–7]. A major drawback associated with these broad-spectrum HDAC inhibitors involves the interaction with all human Zn^2+^-dependent HDACs, culminating in a higher risk to elicit toxic side effects upon administration. Therefore, the selective inhibition of *pf*HDACs over *h*HDAC isoforms represents a relevant challenge in antimalarial drug discovery and has led to the assessment of many *h*HDAC inhibitors as potential antiplasmodial agents. In that respect, a library screen of 2000 compounds has revealed (*E*)-7-[2-(2-bromobenzylidene)hydrazinyl]-*N*-hydroxy-7-oxoheptanamide to be such a selective compound and, in another report, a specific class of methylamides has been shown to be *pf*HDAC selective [12,13].

An alternative strategy could imply the examination of selective *h*HDAC inhibitors (instead of pan-*h*HDAC inhibitors) as novel antimalarial compounds. This approach lowers the risk of host toxicity without potentially compromising a pronounced antiplasmodial activity [14]. Selective human HDAC6 inhibitors could possibly serve this goal as it is known that mice lacking HDAC6 develop rather normally [15], so minor to no side effects are expected upon deployment of these agents. Bearing this rationale in mind, we decided to test a series of benzohydroxamic acids (previously developed by us) for their antiplasmodial activity, with several representatives being highly potent and selective *h*HDAC6 inhibitors. Indeed, a systematic exploration of the possible correlation between *h*HDAC6 inhibitors and antiplasmodial activity has not been performed so far and could reveal new opportunities in antimalarial drug development.

## Materials & methods

### Antiplasmodial assay

Continuous *in vitro* cultures of asexual erythrocyte stages of *P. falciparum* were maintained using a modified method of Trager and Jensen [16]. Quantitative assessment of antiplasmodial activity *in vitro* was determined via the parasite lactate dehydrogenase assay using a modified method described by Makler [17]. The test samples were prepared to a 20 mg/ml stock solution in 100% DMSO. Stock solutions were stored at -20°C. Further dilutions were prepared in complete medium on the day of the experiment. CQ and artesunate were used as the reference drugs. A full dose–response was performed to determine the concentration inhibiting 50% of parasite growth (IC_50_ value). Test samples were tested at a starting concentration of 100 μg/ml, which was then serially diluted twofold in complete medium to give 10 concentrations; with the lowest concentration being 0.2 μg/ml. The same dilution technique was used for all samples. References were tested at a starting concentration of 1 μg/ml. The highest concentration of solvent to which the parasites were exposed to had no measurable effect on the parasite viability (data not shown).

### MTT assay

Test samples were screened for *in vitro* cytotoxicity against a mammalian cell-line, Chinese Hamster Ovary (CHO), using the 3-(4,5-dimethylthiazol-2-yl)-2,5-diphenyltetrazoliumbromide (MTT)-assay. The MTT-assay is used as a colorimetric assay for cellular growth and survival, and compares well with other available assays [18,19]. The tetrazolium salt MTT was used to measure all growth and chemosensitivity. The test samples were tested in triplicate on one occasion. The same stock solutions prepared for antiplasmodial evaluation were used for cytotoxicity testing. Test compounds were stored at -20°C until use. Dilutions were prepared on the day of the experiment. Emetine was used as the reference drug in all experiments. The starting concentration was 100 μg/ml, which was serially diluted in complete medium with tenfold dilutions to give six concentrations, the lowest being 0.001 μg/ml. The highest concentration of solvent to which the cells were exposed to had no measurable effect on the cell viability (data not shown). The IC_50_ values were obtained from full dose–response curves, using a nonlinear dose–response curve fitting analysis via GraphPad Prism v.4 software.

## Results & discussion

This brief article focuses on the antiplasmodial evaluation of three innovative classes of benzohydroxamic acids **1–3**, all featuring a different thiahetero(bi- or tri-)cyclic ‘cap group’ (Figure 1). Class **1** consists of molecules containing a saturated thiaheterocyclic ring annulated onto an indole core (designated as Tubathians), class **2** comprises benzothiophenes embodying a nitrogen atom in the linker region and class **3** includes cycloalkane-annulated 1,5-benzothiazepine scaffolds. Because of small structural modifications with respect to the ‘mother structure’ within each class (Figure 1 & Table 1), a broad set of 42 compounds with divergent decoration patterns is synthetically available. The preparation of these compounds **1–3** has previously been described, together with a detailed account on their HDAC6 selectivity, cellular activity (α-tubulin acetylation, a known substrate of HDAC6) and mutagenicity [20–23]. For Tubathian structures **1**, additional information concerning the ADME/Tox properties has been disclosed as well [22]. These different classes include a number of highly potent and selective *h*HDAC6 inhibitors (Table 2), which have in common a *para*-substituted benzohydroxamic acid fragment, no substituents in the *meta*-position with respect to the hydroxamic acid group, and superior HDAC6 inhibitory activity for sulfoxides and sulfones over the corresponding sulfides.

The antiplasmodial activity of this set of structures was first determined through a modified parasite lactate dehydrogenase assay against a CQ-sensitive (CQS) strain of *P. falciparum* (NF54) [16,17]. When the molecules proved to be reasonably active against this strain (IC_50_ <5 μM), a second assay was performed against a CQ-resistant (CQR) strain of *P. falciparum* (Dd2), as well as an MTT-assay (3-(4,5-dimethylthiazol-2-yl)-2,5-diphenyltetrazolium bromide) on CHO cells to assess their mammalian cytotoxicity (Table 2) [18]. Finally, a selectivity index (SI = IC_50_ CHO/IC_50_ NF54) and a resistance index (RI = IC_50_ Dd2/IC_50_ NF54) was calculated to be able to easily compare the therapeutic window (active concentration vs toxic concentration) and sensitivity toward resistance developing. Table 2 shows that all 42 benzohydroxamic acids **1–3** display interesting antiplasmodial activities (IC_50_ values against the CQS strain between 0.11 and 37.5 μM). The potent HDAC6 inhibitors **1i**, **1k**, **1l**, **3d**, **3e** and **3f** were also found to be highly active against both CQS and CQR parasitic strains (IC_50_ CQS and CQR < 1 μM, IC_50_ HDAC6 < 0.07 μM). However, other active HDAC6 inhibitors did not demonstrate a distinct submicromolar parasitic toxicity (e.g., **1a**, **1e** and **1g**). Thus, no consistent correlation can be drawn between *h*HDAC6 inhibition and antiplasmodial activity, which could be expected considering the inevitable differences between human and parasite HDAC isoforms [11]. On the other hand, it is remarkable to note that the most effective antiplasmodial compounds all are powerful *h*HDAC6 inhibitors, and none of the less active *h*HDAC6 inhibitors showed submicromolar antiplasmodial potency. Based on these observations, it can be suggested that strong *h*HDAC6 inhibitory activity is a necessary but not a sufficient condition for thiaheterocyclic benzohydroxamic acids to exert submicromolar antiplasmodial activity as well. From the six compounds showing the most promising antiplasmodial activity, four molecules have excellent selectivity indices higher than 300 (**1i**, **1k**, **1l** and **3d**), which means that the concentration at which they kill the parasite is at least 300-times lower than their toxic concentration for CHO cells. Comparison of the resistance indices (RI) suggests that the tested molecules have comparable activity (RI = 0.3–3.9) against both strains (CQS and CQR). This is in marked contrast to the control drug CQ, which is 17-times less active against the CQR strain (RI = 17.5).

## Conclusion

42 thiaheterocyclic benzohydroxamic acids **1–3**, 17 of them previously being identified as highly potent and selective *h*HDAC6 inhibitors, were assessed in terms of their antiplasmodial profile. This study revealed six selective HDAC6 inhibitors to demonstrate submicromolar antiplasmodial potency against both a CQS and a CQR strain, and four of these structures (**1i**, **1k**, **1l** and **3d**) also proved to have an excellent therapeutic window (SI > 300). On the other hand, hydroxamic acids which do not strongly inhibit *h*HDAC6, appear to possess only moderate antiplasmodial effects. Thus, potent and selective *h*HDAC6 inhibitory activity of thiaheterocyclic benzohydroxamic acids seems to be a necessary but not a sufficient condition to elicit pronounced antiplasmodial activity as well. Moreover, selective *h*HDAC6 inhibitors can induce powerful *P. falciparum* toxicity without being toxic for CHO cells (as a model for mammalian cytotoxicity). In conclusion, *h*HDAC6 inhibitory activity and antiplasmodial activity are somehow interconnected, and these HDAC6i new chemical entities can certainly be considered a valuable starting point for further medicinal chemistry investigation *en route* to novel types of antiplasmodial drugs.

## Future perspective

The evaluation of isoform-selective *h*HDAC inhibitors, in particular *h*HDAC6is, as antiplasmodial agents represents a new and promising approach in antimalarial research, and further study is desirable to unravel the specific interplay between these compounds and their antiplasmodial activity (structure–activity relationship). In addition to this phenotypic approach, more information on the structure and function of *pf*HDACs should be obtained, which will enable rational design of *pf*HDAC inhibitors.

**Table 1.** **Substitution pattern of thiaheterocyclic benzohydroxamic acids 1–3.**

**Compound**	**R^1^**	**R^2^**	**R^3^**	**x**	**n**	**Config.^†^**
**1a**	H	H	–	0	1	*Para*

**1b**	H	OMe	–	0	1	*Para*

**1c**	F	H	–	0	1	*Para*

**1d**	F	OMe	–	0	1	*Para*

**1e**	H	H	–	2	1	*Para*

**1f**	H	OMe	–	2	1	*Para*

**1g**	F	H	–	2	1	*Para*

**1h**	F	OMe	–	2	1	*Para*

**1i**	Br	H	–	2	1	*Para*

**1j**	H	H	–	1	1	*Para*

**1k**	F	H	–	1	1	*Para*

**1l**	H	H	–	2	0	*Para*

**1m**	F	H	–	2	0	*Para*

**1n**	H	H	–	0	1	*Meta*

**1o**	F	H	–	0	1	*Meta*

**1p**	H	H	–	2	1	*Meta*

**1q**	F	H	–	2	1	*Meta*

**1r**	Ph	H	–	2	1	*Meta*

**1s**	H	H	–	2	0	*Meta*

**1t**	F	H	–	2	0	*Meta*

**1u**	Br	H	–	1	0	*Meta*

**2a**	H	H	H	–	–	–

**2b**	H	Bn	H	–	–	–

**2c**	5-Br	H	H	–	–	–

**2d**	5-Br	Bn	H	–	–	–

**2e**	5-Ph	H	H	–	–	–

**2f**	5-Ph	Bn	H	–	–	–

**2g**	6-Br	H	H	–	–	–

**2h**	6-Br	Bn	H	–	–	–

**2i**	6-Ph	H	H	–	–	–

**2j**	6-Ph	Bn	H	–	–	–

**2k**	H	H	Me	–	–	–

**3a**	H	–	–	0	1	*Cis*

**3b**	Cl	–	–	0	1	*Cis*

**3c**	CF_3_	–	–	0	1	*Cis*

**3d**	H	–	–	2	1	*Cis*

**3e**	Cl	–	–	2	1	*Cis*

**3f**	CF_3_	–	–	2	1	*Cis*

**3g**	H	–	–	1	1	*Cis*

**3h**	H	–	–	0	2	*Cis*

**3i**	Cl	–	–	0	1	*Trans*

**3j**	H	–	–	0	2	*Trans*

^†^The *para*- and *meta*-configuration for compound **1** refers to the position of the hydroxamic acid group on the aromatic ring with respect to the aminomethyl substituent.

**Table 2.** **IC_50_ values (μM) of compounds 1–3 determined for a normal (NF54) and chloroquine-resistant (Dd2) *Plasmodium falciparum* strain, Chinese hamster ovary cells and HDAC6.**

**Cmpd**	**NF54**	**Dd2**	**CHO**	**SI^‡^**	**RI^§^**	**HDAC6**
**1a**	37.5	–	–	–	–	0.015

**1b**	14.0	–	–	–	–	–

**1c**	2.2	3.1	105.2	48	1.4	0.022

**1d**	23.2	–	–	–	–	–

**1e**	10.8	–	–	–	–	0.002

**1f**	21.0	–	–	–	–	2.0

**1g**	15.8	–	–	–	–	0.004

**1h**	32.7	–	–	–	–	1.3

**1i**	**0.11**	**0.43**	**109.0**	**991**	**3.9**	**0.003**

**1j**	1.28	1.3	>282	>217	1.0	0.014

**1k**	**0.40**	**0.80**	**>269**	**>673**	**2.0**	**0.009**

**1l**	**0.92**	**0.66**	**>281**	**>305**	**0.7**	**0.008**

**1m**	1.07	1.55	>267	>250	1.7	0.016

**1n**	1.48	2.18	>295	>199	1.5	–

**1o**	1.32	1.44	48.1	36	1.1	–

**1p**	5.45	–	–	–	–	–

**1q**	7.84	–	–	–	–	–

**1r**	8.13	–	–	–	–	–

**1s**	12.2	–	–	–	–	–

**1t**	9.80	–	–	–	–	–

**1u**	11.9	–	–	–	–	–

**2a**	1.60	2.13	12.7	8	1.3	0.014

**2b**	32.4	–	–	–	–	–

**2c**	1.02	2.4	31.0	30	2.4	0.037

**2d**	17.8	–	–	–	–	–

**2e**	5.07	–	–	–	–	–

**2f**	5.75	–	–	–	–	–

**2g**	1.30	1.58	46.1	35	1.2	0.064

**2h**	8.45	–	–	–	–	–

**2i**	3.34	1.14	31.2	9	0.3	–

**2j**	5.02	–	–	–	–	–

**2k**	36.8	–	–	–	–	–

**3a**	1.59	>2.7	103.9	65	–	0.036

**3b**	>2.48	>2.48	41.4	–	–	0.650

**3c**	1.53	>2.29	61.5	40	–	0.200

**3d**	**0.36**	**0.94**	**107.6**	**303**	**2.6**	**0.008**

**3e**	**0.47**	**0.44**	**35.4**	**75**	**0.9**	**0.068**

**3f**	**0.87**	**0.70**	**56.8**	**65**	**0.8**	**0.011**

**3g**	1.25	>2.60	172.9	138	–	0.006

**3h**	>2.61	>2.61	87.0	–	–	0.033

**3i**	>2.48	1.57	50.9	–	–	0.160

**3j**	>2.61	>2.61	61.7	–	–	0.092

Bold: IC_50_-value of the hydroxamic acid lower than 1 μM against both *P. falciparum* strains. ‘–’ is not determined.

^‡^SI (selectivity index) = IC_50_ (CHO)/IC_50_ (NF54).

^§^RI (resistance index) = IC_50_ (Dd2)/IC_50_ (NF54).

Chloroquine IC_50_-NF54 = 0.01 μM, IC_50_-Dd2 = 0.175 μM; Artesunate IC_50_-NF54 < 0.01 μM, IC_50_-Dd2 = 0.016 μM; Emetine IC_50_-CHO = 0.112 μM.

CHO: Chinese hamster ovary; RI: Resistance index; SI: Selectivity index.

**Executive summary**

- Antimalarial drug resistance encourages the need for innovative chemical entities with alternative modes of action.
- *h*HDAC6 inhibitors are proposed as a novel strategy against *Plasmodium falciparum*.

**Results**

- Three classes of thiaheterocyclic benzohydroxamic acids were tested against *P. falciparum*.
- Four potent and selective *h*HDAC6 inhibitors were shown to be highly active as antiplasmodial agents, displaying a good therapeutic window and resistance index as well.

**Conclusion**

- Potent and selective *h*HDAC6 inhibitory activity of thiaheterocyclic benzohydroxamic acids is a necessary but not a sufficient condition to elicit pronounced antiplasmodial activity.
- *h*HDAC6 inhibitors can induce powerful *P. falciparum* toxicity without being toxic for CHO cells.
